# Differential response of the liver to bile acid treatment in a mouse model of Niemann-Pick disease type C

**DOI:** 10.12688/wellcomeopenres.12431.2

**Published:** 2018-04-04

**Authors:** Elena-Raluca Nicoli, Mylene Huebecker, David Smith, Lauren Morris, Frances M. Platt

**Affiliations:** 1 Department of Pharmacology, University of Oxford, Oxford, OX1 3QT, UK

**Keywords:** Niemann Pick disease type C, NPC1, lysosomal storage disease, p450, bile acids, ursodeoxycholic acid, cholic acid, liver

## Abstract

Niemann-Pick disease type C (NPC) disease is a neurodegenerative lysosomal storage disease caused by mutations in the *NPC1* or *NPC2* genes. Liver disease is also a common feature of NPC that can present as cholestatic jaundice in the neonatal period. Liver enzymes can remain elevated above the normal range in some patients as they age.

We recently reported suppression of the P450 detoxification system in a mouse model of NPC disease and also in post-mortem liver from NPC patients. We demonstrated the ability of the hydrophobic bile acid ursodeoxycholic acid (UDCA) (3α, 7β-dihydroxy-5β-cholanic acid) to correct the P450 system suppression. UDCA is used to treat several cholestatic disorders and was tested in NPC due to the P450 system being regulated by bile acids. Here, we compare the effect of UDCA and cholic acid (CA), another bile acid, in the NPC mouse model. We observed unexpected hepatotoxicity in response to CA treatment of NPC mice. No such hepatotoxicity was associated with UDCA treatment. These results suggest that CA treatment is contraindicated in NPC patients, whilst supporting the use of UDCA as an adjunctive therapy in NPC patients.

## Introduction

Niemann-Pick disease type C (NPC) is a rare progressive neurodegenerative lysosomal disorder, which affects 1:100,000 live births ^1, 2^. In addition to the central nervous system, the liver also plays a role in this disease, either presenting in infancy as cholestatic jaundice or if these patients survive as elevated levels of liver enzymes suggestive of chronic liver dysfunction ^1^. Previous published studies of murine *Npc1* ^*-/-*^ models identified other treatment modalities for hepatic dysfunction. These included the cholesterol absorption inhibitor, ezetimibe, and 2-hydroxypropyl-β-cyclodextrin (2HPβCD) ^3^.

We recently discovered that the hepatic cytochrome P450 system is suppressed in the *Npc1* null mouse model ( *Npc1 ^-/-^),* in the feline model of NPC disease and in three NPC human liver samples (post-mortem) ^4^. In NPC, the efflux of unesterified cholesterol from late endosomes/lysosomes to the endoplasmic reticulum (ER) is inhibited, leading to a shift in the spectrum of bile acids synthesized ^5, 6^, which suppresses gene expression of the P450 system ^4^. Treatment of *Npc1 ^-/-^* mice with ursodeoxycholic acid (UDCA; 3α, 7β-dihydroxy-5β-cholanic acid) rescued gene expression and P450 system enzyme activity ^4^. In this study, we compared the effects of UDCA (soluble bile acid) and cholic acid (CA; hydrophobic bile acid) in the *Npc1 ^-/-^* mouse and discovered hepatotoxic adverse effects on the liver in response to CA, but not to UDCA, suggesting UDCA is the bile acid of choice for clinical treatment of NPC patients.

## Methods

### Npc1 mouse model

*Npc1* mutant (BALB/cNctr- *Npc1m1N*/J, *Npc1* ^-/-^) and control ( *Npc1* ^+/+^) were generated from heterozygote breeding. Genotyping was performed as described ^7^ and mixed sex groups analysed. All mice were maintained under a standard 12h light/12h dark cycle with water and food available *ad libitum*. All procedures were performed according to the Animals (Scientific Procedures) Act 1986 under a project license (PPL No. 30/2923) from the UK Home Office. Care was taken to minimise suffering through euthanizing the animals once liver enlargement was unambiguously detected by visual inspection (i.e. 3 weeks after initiation of treatment).

### Bile acid supplementation

*Npc1 ^-/-^* and *Npc1 ^+/+^* mice (n = 9-17 per group) were fed either normal chow (RM1 maintenance diet; SDS, London, UK) or normal chow supplemented with UDCA (0.5%, w/w, Sigma-Aldrich) or CA (0.5%, w/w, Sigma-Aldrich) mixed with powdered diet. Treatment started at weaning (3 weeks of age) and mice were sacrificed at 6 weeks of age by intraperitoneal injection with an overdose of phenobarbital.

### Quantification of cholesterol

Cholesterol was quantified using an Amplex Red Cholesterol Assay Kit (Thermo Fisher), according to manufacturer’s instructions.

### Histology

Animals were euthanized at 6 weeks of age and were transcardiac perfused with 4% paraformaldehyde (PFA); samples of liver were fixed in 4% PFA, dehydrated and processed to wax using standard procedures. The wax blocks were cut at 4-μm-thick sections, mounted on SuperFrost Plus slides (Thermo Scientific, Waltham, MA, USA) and subjected to hematoxylin and eosin (H&E) staining by standard methods. Images were taken on a Nikon eclipse E200 microscope with a Leica digital camera system.

### Statistical analysis

**Figure 1.**
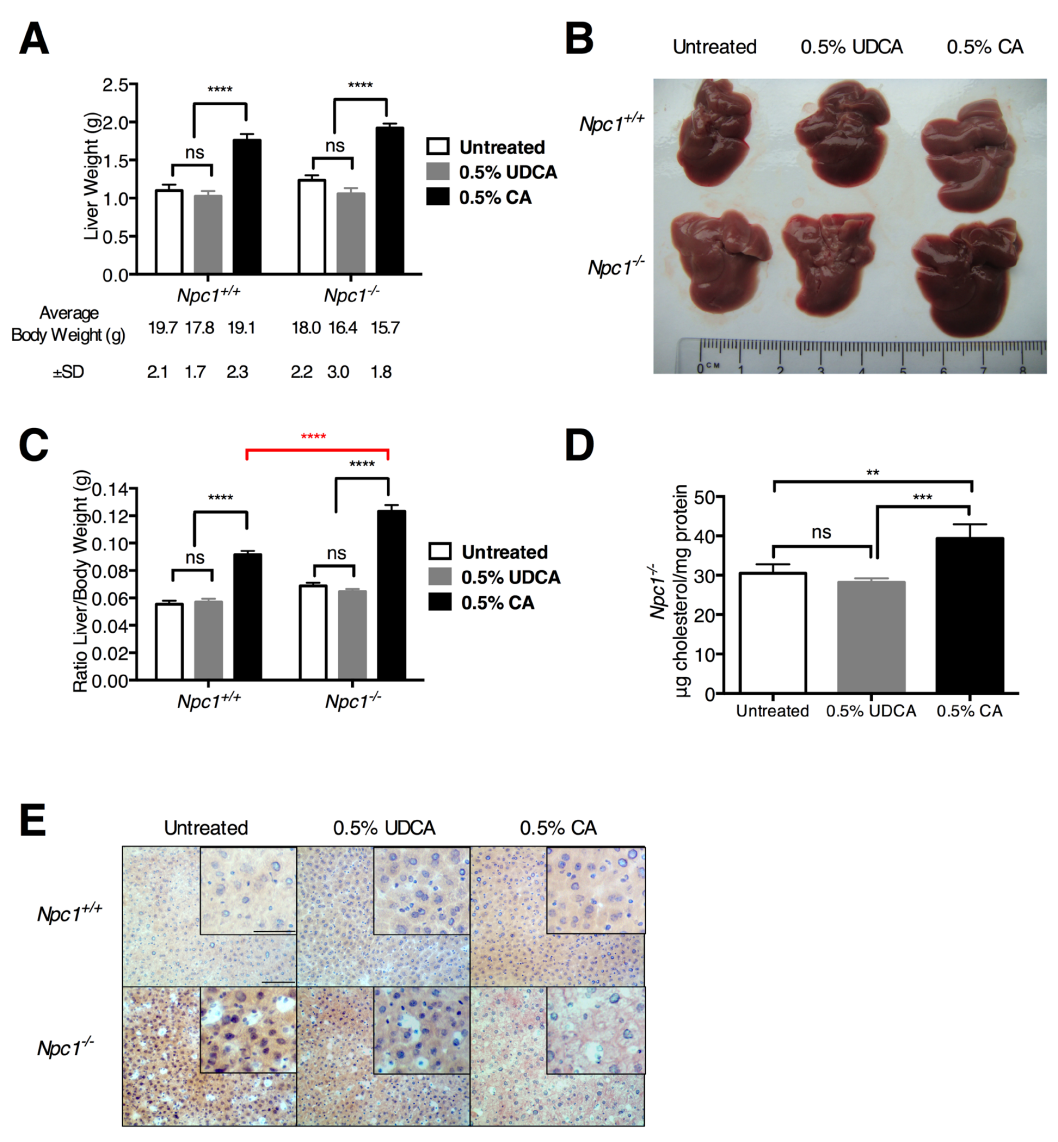
Effects of cholic acid (CA) and ursodeoxycholic acid (UDCA) on 6-week old *Npc1* mouse liver. *Npc1 ^+/+^* and *Npc1* ^-/-^ mice were treated with 0.5% UDCA or CA as admix with powdered diet from three weeks of age. The mice were euthanized at 6 weeks of age when the *Npc1* ^-/-^ mice exhibited abdominal distension due to liver enlargement. ( **A**) Liver weights of *Npc1* ^-/-^ mice untreated, treated with 0.5% UDCA and 0.5% CA; average body weight ± SD of the mice are shown beneath each bar for each group. ( **B**) Gross morphology of the liver. ( **C**) Ratio of liver weight to mouse body weight. Data are presented as mean ± SEM, n = 9–17 animals per group/genotype, **** *p*< 0.0001 calculated using two-way ANOVA with Tukey’s multiple comparison test. Red line indicates comparison between CA treated wild-type and *Npc1* ^-/-^ mice. ( **D**) Quantification of cholesterol levels in the liver of untreated, UDCA-treated and CA-treated *Npc1* ^*-/-*^ mice. Values are adjusted for sample protein concentration. Data are presented as mean ± SEM, n= 3–4 livers per group, ** *p*< 0.01, *** *p*< 0.001 calculated using one-way ANOVA with Tukey’s multiple comparison test. ( **E**) H&E histopathology of liver sections, bars represent 10μm for high magnification inset panels and 5μm for the main panel.

Two-way ANOVA with Tukey’s multiple comparison was used to analyse the sets of data comparing *Npc1 ^+/+^* and *Npc1 ^-/-^* mice for Figure 1 A and C. One-way ANOVA with Tukey’s multiple comparison was used to analyse the data comparing the treatment groups in *Npc1* ^-/-^ mice in Figure 1D. Statistical analysis was performed with GraphPad Prism version 6.

## Results

We investigated the effects of UDCA and CA treatment in the *Npc1 ^-/-^* mouse model, relative to wild type ( *Npc1 ^+/+^*) mice. Mice were fed on a diet containing either 0.5% UDCA or CA from weaning (3 weeks of age). After 3 weeks of treatment there was gross abdominal distention observed in all CA treated mice, irrespective of genotype, but the degree of distension appeared greater in the *Npc1 ^-/-^* mice. No distension of the abdomen was observed in UDCA treated *Npc1 ^-/-^* and *Npc1 ^+/+^* mice. On necropsy, it was apparent that the abdominal distension in the CA treated group was due to liver enlargement ( Figures 1A and B), and there was a statistically significant increase of liver weight in the *Npc1 ^+/+^* and *Npc1 ^-/-^* relative to UDCA and untreated mice ( *p*<0.0001). No significant changes in liver volume were observed in the UDCA treated mice irrespective of genotype ( Figures 1A and B). *Npc1 ^-/-^* mice have a lower body weight than wild type control mice ^8^, and so the ratio of liver/body weight was calculated ( Figure 1C). The liver enlargement in response to CA as a function of body weight was greater in the *Npc1 ^-/-^* than in the *Npc1 ^+/+^* mice. The liver-to-body weight ratios were the same in untreated *Npc1 ^+/+^* and *Npc1 ^-/-^* mice and in the UDCA treated mice, irrespective of genotype. Bile acids have previously been shown to affect intestinal absorption of cholesterol, with UDCA and CA demonstrated to decrease and increase absorption respectively ^9^. Quantification of liver cholesterol levels revealed that cholesterol levels in the UDCA treated mice were unchanged relative to untreated *Npc1* ^*-/-*^ ( *p*=0.4025), whilst CA treatment was associated with a statistically significant ( *p*<0.01) increase (29.2%) in liver cholesterol relative to the untreated group. ( Figure 1D).

Histopathological analysis of liver ( Figure 1E) showed the typical vacuolated appearance of the liver characteristic of *Npc1 ^-/-^* mice relative to wild type mice. UDCA treatment had no impact on the histopathology of wild type and *Npc1 ^-/-^* mice, whereas CA treatment led to hepatocyte enlargement and a foamy appearance indicative of lipid accumulation that often displaced the nucleus to the pole of the cell. 

## Discussion

NPC disease cells display a complex cellular pathophysiology, including impaired movement of LDL-derived cholesterol from late endosomes/lysosomes to the ER ^10^ and the generation of non-enzymatically generated oxysterols ^11^. As a consequence, the bile acid profile is altered in NPC disease ^5, 6^, and this change in bile acid composition leads to a secondary suppression of the P450 gene family, as they are bile acid regulated ^4^. The expression of the P450 system genes could be rescued in *Npc1 ^-/-^* mice by administering the bile acid UDCA, leading to clinical benefit ^4^.

In another study (complementary to the murine work detailed here), UDCA was trialed in four clinical NPC cases with improvements in liver function (including reduced aspartate aminotransferase (AST) and alanine transaminase (ALT)) in those patients with elevated liver enzymes at baseline ^12^.

In this study, we investigated whether CA, another bile acid used in routine clinical practice for bile acid disorders ^13^, would also be beneficial. Therefore, we treated *Npc1 ^-/-^* mice with UDCA or CA to investigate this further. However, three weeks into treatment the CA treated mice presented with gross abdominal distension and were subsequently culled to determine the basis for this adverse finding. It was clear upon gross necropsy that the livers were significantly enlarged in CA treated wild type and *Npc1 ^-/-^* mice. When adjusted for body weight it was apparent that the liver enlargement was greater in the *Npc1 ^-/-^* mice relative to wild type mice. CA treated wild type mice had an increased liver volume (post-adjustment for body weight) of 165.5% relative to their untreated counterparts, whereas the CA treated *Npc1 ^-/-^* mice exhibited a 179.1% increase in liver volume when compared with untreated *Npc1 ^-/-^* mice.

Histopathology revealed lipid-laden distended hepatocytes in the CA treated *Npc1 ^-/-^* mice. These data suggest CA is more hepatotoxic in mice than UDCA, consistent with the differential chemical properties of these two bile acids. The NPC1 protein, deficient in most cases of NPC disease, is a conserved member of a protein family, the RND permeases ^14^. In bacterial systems they serve to efflux multiple classes of substrates across the periplasmic space, allowing bacteria to thrive in hostile environments ^15^. CA’s hydrophobic chemistry suggest it may be a potential substrate for NPC1 ^15^, so it may itself be stored in the late endocytic system in *NPC1* deficient cells ** and adversely affect the acidic compartment of the cell leading to further lipid storage. This hypothesis merits further investigation.

When four NPC1 patients were treated with UDCA, benefit was observed based on improved liver function ^12^. Very interestingly, one patient was switched from UDCA to CA and their clinical status declined but was recovered upon switching back to UDCA. This has striking parallels with this murine study.

When considered in light of the accompanying clinical study these data strongly suggest that whereas UDCA has potential as an adjunctive therapy to treat residual liver disease in NPC patients, CA is contraindicated due to acute hepatotoxicity that is most pronounced in an NPC1 deficient background.

## Data availability

Raw data have been uploaded to the online data repository OSF: http://dx.doi.org/10.17605/OSF.IO/5A2F9 ^16^
