# Supplementary material for: Differential response of the liver to bile acid treatment in a mouse model of Niemann-Pick disease type C
Source: Wellcome Open Res. 2018 Apr 4;2:76. Originally published 2017 Aug 31. [Version 2] doi: 10.12688/wellcomeopenres.12431.2 (PMC5639931; doi:10.12688/wellcomeopenres.12431.2)
Supplement: Peer review history

# Peer review: Referee response for version 2

**DOI:** 10.21956/wellcomeopenres.15584.r32737

**Published:** 2018-04-19

**Authors:** Stephen D Turley

**Affiliations:** 1 Southwestern Medical Center, University of Texas, Dallas, TX, USA

It is misleading to say  “---the liver also plays a role in this disease” . What should be said is that “--- although neurodegenerative disease is the principal cause of morbidity and mortality in NPC1 deficiency, pulmonary dysfunction or liver failure are the cause of death in subsets of  NPC1 patients  (add at least three supporting refs here ). While 2-hydroxypropyl-beta-cyclodextrin is so far the most effective agent for managing neurodegeneration in NPC1 patients , in both mouse and feline models of NPC1 disease this compound has been shown also to significantly blunt or reverse hepatic unesterified cholesterol sequestration and dysfunction  (add two ref here, these being Vite et al. in Science Translational Medicine Vol 7  Issue 276  276ra26, 2015  AND Ramirez et al. in Pediatric Research 68: 309-315 , 2010). Other agents evaluated in NPC1 mouse models suggest that ezetimibe, a potent cholesterol absorption inhibitor (Beltroy et al.--- Ref 3  cited in revised submission), and more recently  the HDAC inhibitor, Vorinostat (Munkacsi et al. J Biol Chem 292:4395-4410,2017).

 What the current study by Nicoli et al. is about is essentially to address the question of how changing the intestinal bile acid pool composition  by feeding high levels of two different bile acids may affect liver cholesterol content and health in an NPC1 mouse model. Here it should be stated that, based on the definitive studies of Wang et al (cited in the revised submission), feeding cholic acid (which promotes cholesterol absorption and increases hepatic chol levels) versus ursodeoxycholic acid (which has the opposite effects) to an NPC1 mouse model would hypothetically have very different effects on hepatic cholesterol content and liver health in general. What is shown by Nicoli et al. is that when cholesterol absorption is impeded  in the NPC1 mice by making ursodeoxycholic acid a major BA in their intestines, cholesterol absorption falls, just as it does with very low doses of ezetimibe in the diet of the NPC1 mouse. The opposite happens in NPC1 mice given cholic acid.

I have read this submission. I believe that I have an appropriate level of expertise to confirm that it is of an acceptable scientific standard.

# Peer review: Referee response for version 1

**DOI:** 10.21956/wellcomeopenres.13461.r26337

**Published:** 2017-10-12

**Authors:** Roberto Giugliani

**Affiliations:** 1 National Institute on Population Medical Genetics - INAGEMP, Porto Alegre, Brazil; 2 Department of Genetics, Federal University of Rio Grande do Sul, Porto Alegre, Brazil; 3 Medical Genetics Service, Hospital de Clínicas de Porto Alegre, Porto Alegre, Brazil

This report is based in an interesting experiment, and the conclusions are supported by the data presented. The research problem was adequately addressed, and the paper is well written. The experiment was designed after the discovery that the hepatic cytochrome P450 system is suppressed in NPC animal models and also in NPC human liver (post-mortem analyses). As previously described, the inhibition of the efflux of unesterified cholesterol from the lysosomal system to the endoplasmic reticulum changes the spectrum of bile acids synthesized, and this eventually suppresses gene expression of the P450 system. It was already demonstrated that treatment of the NPC models with ursodeoxycholic acid (UCDA) rescued P450 activity. In this study, therapy with UCDA was compared with therapy with cholic acid (CA), being hepatotoxic adverse effects observed with CA but not with UCDA, which could be further explored as a potential adjunctive therapeutic agent in NPC.

I have read this submission. I believe that I have an appropriate level of expertise to confirm that it is of an acceptable scientific standard.

# Peer review: Referee response for version 1

**DOI:** 10.21956/wellcomeopenres.13461.r26339

**Published:** 2017-10-02

**Authors:** Stephen D Turley

**Affiliations:** 1 Southwestern Medical Center, University of Texas, Dallas, TX, USA

Although the rationale for this study is clearly explained, the relevance of the data obtained to the management of liver disease in NPC1 deficiency is questionable for reasons relating mainly but not fully to the methodological approaches that were used. The following points warrant close consideration.

1. Although the dietary level of BA supplementation employed is in line with what is often used in animal models, the changes in bile acid pool and composition that ensue when the amount of dietary BA is set at 0.5% w/w are beyond extreme and result in shifts in cholesterol metabolism at multiple levels that would never occur in humans taking approved doses of any specific BA. At a dietary level of 0.5%,  the dose used in these studies was the equivalent of around 800 mg/day /kg bw which far exceeds what most human subjects taking oral BAs consume during a 24 hour period.  When BA pool size and composition change in extreme ways there are invariably marked shifts in intestinal cholesterol absorption and this in turn has a major impact on the amount of chylomicron cholesterol reaching the liver from the small intestine (and therefore in the amounts of unesterified chol –UC—becoming subsequently trapped in the late /E/L compartment when NPC1 or NPC2 are mutated). There is an excellent study by Wang *et al.* (2003) ^1^ showing how a range of BAs including UDCA and CA  (added to the diet at a level of 0.5%) affect intestinal cholesterol absorption in a normal mouse model (see Fig 3 in particular). While a BA pool enriched with UDCA drives down intestinal cholesterol absorption, the opposite effect occurs with CA. Another example illustrating how BA pool size and composition are major determinants of cholesterol absorption is seen in a study by Schwarz *et al.* (1998) ^2^ –see solid histograms for WT mice given hyodeoxycholic acid vs cholic acid  in Fig 5. Anyone who knows about the properties of CA and who also understands the biochemical consequences of NPC1 deficiency would view the effects of CA described in the current report as “totally predictable” especially given the massive dose that was fed to the mice. It would have been better to use two different doses of UDCA.
2. The bottom line in all this is that the differing impacts on liver “health” of UDCA vs CA supplementation in NPC1 ^-/- ^mice shown here are likely fully explainable on the basis of major differences in the amount of intestinally derived cholesterol  trapped in the livers of the KOs given these two particular BAs. Although no data are presented for hepatic total chol concs (mg/g), one might predict from other studies in the NPC1 mouse model that a several fold greater level of unesterified chol (UC) would be seen in the livers of the CA supplemented mice vs those given UDCA. There is no question that it is the cytotoxic effects of the entrapped UC in all organs, especially the liver, that has the most damaging impact in NPC1 (or NPC2) dysfunction.
  While it is recognized that this is a preliminary report, it is essential that more informed conclusions be reached about what UDCA supplementation might conceivably be doing in the human NPC1-deficient liver. To achieve this, definitely the liver UC and EC concentrations in these mice, along with their plasma/serum ALT and AST levels should be measured. Such measurements  are needed if these mouse model experiments are to provide any credible guide as to how UDCA supplementation in human NPC1 patients might be acting. The benefit most likely has nothing to do with putative improvement in functionality of the P450 detoxification system.
3. As it is currently written, this report will likely attract criticism because of its lack of acknowledgement of  significant published work documenting the beneficial impact of other types of treatment modalities for improving liver function in NPC1 deficiency,  mostly based on *in vivo *experiments using primarily the NPC1 ^-/- ^mouse model. In 2007 Beltroy *et al.* showed that the cholesterol absorption inhibitor, ezetimibe, given  in the diet from the time of weaning, at doses far below those of other treatments (ie 20 or less mg /day/kg bw), reduced hepatomegaly and lowered liver cholesterol content (nearly all UC) by almost 50% (Fig 4) ^3^. The benefit of ezetimibe in this disease is largely in the liver. After that, a series of definitive publications from a number of labs attesting the potency of subcutaneously administered 2HPBCD in diminishing hepatic UC content and markedly improving liver function tests (and increasing lifespan) in NPC1 mouse models helped establish this agent as one of great potential for managing multisystem disease in NPC1 deficiency ^4^^,^^5^.

Although this preliminary report will be of interest to many working in the field of NPCD management, it should not generate the impression that UDCA has a clear potential to become a treatment modality for liver disease in NPCD patients. Citation of the earlier papers noted above  documenting the decisive benefits of ezetimibe and 2HPBCD in  NPC1 mice will allow readers to gain a better perspective of what these initial data in the UDCA treated NPC1 mouse model are telling us.

I have read this submission. I believe that I have an appropriate level of expertise to state that I do not consider it to be of an acceptable scientific standard, for reasons outlined above.

## References

[1] WangDQTazumaSCohenDECareyMC: Feeding natural hydrophilic bile acids inhibits intestinal cholesterol absorption: studies in the gallstone-susceptible mouse. *Am J Physiol Gastrointest Liver Physiol*.2003;285(3) : 10.1152/ajpgi.00156.2003 G494-502 10.1152/ajpgi.00156.2003 12748061

[2] SchwarzMRussellDWDietschyJMTurleySD: Marked reduction in bile acid synthesis in cholesterol 7alpha-hydroxylase-deficient mice does not lead to diminished tissue cholesterol turnover or to hypercholesterolemia. *J Lipid Res*.1998;39(9) :1833-43 9741696

[3] BeltroyEPLiuBDietschyJMTurleySD: Lysosomal unesterified cholesterol content correlates with liver cell death in murine Niemann-Pick type C disease. *J Lipid Res*.2007;48(4) : 10.1194/jlr.M600488-JLR200 869-81 10.1194/jlr.M600488-JLR200 17220530

[4] LiuB: Therapeutic potential of cyclodextrins in the treatment of Niemann-Pick type C disease. *Clin Lipidol*.2012;7(3) : 10.2217/clp.12.31 289-301 10.2217/clp.12.31 25152773PMC4138883

[5] DavidsonCDFishmanYIPuskásISzemánJSohajdaTMcCauliffLASikoraJStorchJVanierMTSzenteLWalkleySUDobrenisK: Efficacy and ototoxicity of different cyclodextrins in Niemann-Pick C disease. *Ann Clin Transl Neurol*.2016;3(5) : 10.1002/acn3.306 366-80 10.1002/acn3.306 27231706PMC4863749

# Response

**Published:** 2018-03-22

**Authors:** Fran Platt

**Affiliations:** University of Oxford, UK

Although the rationale for this study is clearly explained, the relevance of the data obtained to the management of liver disease in NPC1 deficiency is questionable for reasons relating mainly but not fully to the methodological approaches that were used. The following points warrant close consideration.

1. Although the dietary level of BA supplementation employed is in line with what is often used in animal models, the changes in bile acid pool and composition that ensue when the amount of dietary BA is set at 0.5% w/w are beyond extreme and result in shifts in cholesterol metabolism at multiple levels that would never occur in humans taking approved doses of any specific BA. At a dietary level of 0.5%, the dose used in these studies was the equivalent of around 800 mg/day /kg bw which far exceeds what most human subjects taking oral BAs consume during a 24 hour period.  When BA pool size and composition change in extreme ways there are invariably marked shifts in intestinal cholesterol absorption and this in turn has a major impact on the amount of chylomicron cholesterol reaching the liver from the small intestine (and therefore in the amounts of unesterified chol –UC—becoming subsequently trapped in the late /E/L compartment when NPC1 or NPC2 are mutated). There is an excellent study by Wang *et al.* (2003) 1 showing how a range of BAs including UDCA and CA  (added to the diet at a level of 0.5%) affect intestinal cholesterol absorption in a normal mouse model (see Fig 3 in particular). While a BA pool enriched with UDCA drives down intestinal cholesterol absorption, the opposite effect occurs with CA. Another example illustrating how BA pool size and composition are major determinants of cholesterol absorption is seen in a study by Schwarz *et al.* (1998) 2 –see solid histograms for WT mice given hyodeoxycholic acid vs cholic acid  in Fig 5. Anyone who knows about the properties of CA and who also understands the biochemical consequences of NPC1 deficiency would view the effects of CA described in the current report as “totally predictable” especially given the massive dose that was fed to the mice. It would have been better to use two different doses of UDCA.

*We thank the reviewer for their comments. We have dealt with comment 1 & 2 together, with our response given below.*

 

1. The bottom line in all this is that the differing impacts on liver “health” of UDCA vs CA supplementation in NPC1-/- mice shown here are likely fully explainable on the basis of major differences in the amount of intestinally derived cholesterol trapped in the livers of the KOs given these two particular BAs. Although no data are presented for hepatic total chol concs (mg/g), one might predict from other studies in the NPC1 mouse model that a several fold greater level of unesterified chol (UC) would be seen in the livers of the CA supplemented mice vs those given UDCA. There is no question that it is the cytotoxic effects of the entrapped UC in all organs, especially the liver, that has the most damaging impact in NPC1 (or NPC2) dysfunction.While it is recognized that this is a preliminary report, it is essential that more informed conclusions be reached about what UDCA supplementation might conceivably be doing in the human NPC1-deficient liver. To achieve this, definitely the liver UC and EC concentrations in these mice, along with their plasma/serum ALT and AST levels should be measured. Such measurements are needed if these mouse model experiments are to provide any credible guide as to how UDCA supplementation in human NPC1 patients might be acting. The benefit most likely has nothing to do with putative improvement in functionality of the P450 detoxification system.

 

*We thank the reviewer for their helpful comments. With regards to a more thorough investigation of liver function we refer the reviewer to a publication (to which this mouse study acts as a supplement) that investigates the effect of UDCA in a small number of NPC patients. The Evans et al clinical case report investigated the effects of UDCA on liver function in NPC patients* ^1^ *. Treatment with UDCA (at between 10 and 30mg/kg/day) was associated with a marked improvement in liver function parameters (including* *aspartate aminotransferase (AST) and alanine transaminase (ALT)* *) in patients with elevated liver enzyme levels at baseline* *. We judged that the existence of human data demonstrating the benefit of therapeutic doses of UDCA on NPC patient liver function obviated the need for extended murine studies. The separation of murine and human studies into separate publications was an editorial requirement of Wellcome Open Research. However, the two studies should be considered together.*

*Importantly, this NPC patient case study (Evans et al) also suggested that cholic acid is deleterious in NPC patients* ^1^ *. One of the patients detailed in the case study was commenced on UDCA from 4 weeks of age due to cholestatic jaundice of uncertain aetiology. A bile acid profile at 2 months of age indicated a potential bile acid synthesis defect, and cholic acid treatment was initiated (at 4.5 months) with the aim of supressing bile acid synthesis. CA treatment was associated with elevations in* *aspartate aminotransferase (AST), alanine transaminase (ALT), alkaline phosphatase, bilirubin and total cholesterol levels. Upon discontinuation of CA and resumption of UDCA treatment there was quantitative improvement in multiple parameters of liver function.*

*We have additional data demonstrating that the beneficial effect of UDCA is not due to effects on liver cholesterol levels. There is no significant difference in the levels of liver cholesterol in the UDCA-treated Npc1 ^-/-^ mouse (relative to untreated Npc1 ^-/-^ mouse, new Figure 1D in the revised manuscript). Whilst CA treatment is associated with a statistically significant elevation in Npc1 ^-/-^ liver cholesterol levels (relative to UDCA-treated Npc1 ^-/-^) the extent of this elevation is less than 1.5 fold. These data have been added to the paper, and the text of the results section expanded accordingly.*

*This study should also be considered in light of our previous published study which fully characterised the beneficial effect of UDCA treatment on body weight, motor function, tremor and was consistent with restoration of the P450 system in Npc1 ^-/-^ mice mediating the therapeutic benefit* ^2^ *.*

1.            Evans, W. R. H., Nicoli, E.-R., Wang, R. Y., Movsesyan, N. & Platt, F. M. Case Report: Ursodeoxycholic acid treatment in Niemann-Pick disease type C; clinical experience in four cases. *Wellcome Open Res.* **2,** 75 (2017).

2.            Nicoli, E.-R. *et al.* Defective Cytochrome P450-Catalysed Drug Metabolism in Niemann-Pick Type C Disease. *PLOS ONE* **11,** e0152007 (2016).

3.    As it is currently written, this report will likely attract criticism because of its lack of acknowledgement of significant published work documenting the beneficial impact of other types of treatment modalities for improving liver function in NPC1 deficiency, mostly based on *in vivo *experiments using primarily the NPC1-/- mouse model. In 2007 Beltroy *et al.* showed that the cholesterol absorption inhibitor, ezetimibe, given  in the diet from the time of weaning, at doses far below those of other treatments (ie 20 or less mg /day/kg bw), reduced hepatomegaly and lowered liver cholesterol content (nearly all UC) by almost 50% (Fig 4) 3. The benefit of ezetimibe in this disease is largely in the liver. After that, a series of definitive publications from a number of labs attesting the potency of subcutaneously administered 2HPBCD in diminishing hepatic UC content and markedly improving liver function tests (and increasing lifespan) in NPC1 mouse models helped establish this agent as one of great potential for managing multisystem disease in NPC1 deficiency 4, 5.

Although this preliminary report will be of interest to many working in the field of NPCD management, it should not generate the impression that UDCA has a clear potential to become a treatment modality for liver disease in NPCD patients. Citation of the earlier papers noted above  documenting the decisive benefits of ezetimibe and 2HPBCD in  NPC1 mice will allow readers to gain a better perspective of what these initial data in the UDCA treated NPC1 mouse model are telling us.

*We thank the reviewer for their comments. We agree that it would be beneficial to acknowledge the published work relating to other types of treatment modalities for hepatic dysfunction in NPC. We have amended the text of the paper accordingly. The intended purpose of this study was to demonstrate that there is a differential response of the Npc1 ^-/- ^mouse to treatment with UDCA and CA, despite both bile acids being widely used in the treatment of cholestatic disorders. CA treatment was deleterious in both Npc1 ^-/-^ mice and NPC patients* ^1^ *and hence is contraindicated for NPC1 ^-/-^.*

*We would suggest, based on observed benefits to hepatic and neurological function (from this publication, the accompanying publication relating to NPC patients and previously published data with Npc1 ^-/-^ mice), that UDCA has the potential to be a valuable addition to combination therapy for NPC* ^1,2^ *.*

1.            Evans, W. R. H., Nicoli, E.-R., Wang, R. Y., Movsesyan, N. & Platt, F. M. Case Report: Ursodeoxycholic acid treatment in Niemann-Pick disease type C; clinical experience in four cases. *Wellcome Open Res.* **2,** 75 (2017).

2.            Nicoli, E.-R. *et al.* Defective Cytochrome P450-Catalysed Drug Metabolism in Niemann-Pick Type C Disease. *PLOS ONE* **11,** e0152007 (2016).

# Peer review: Referee response for version 1

**DOI:** 10.21956/wellcomeopenres.13461.r25792

**Published:** 2017-09-19

**Authors:** Md Suhail Alam

**Affiliations:** 1 Boler-Parseghian Center for Rare and Neglected Diseases, Department of Biological Sciences, University of Notre Dame, Notre Dame, IN, USA

The article by Nicoli *et al* entitled **“Differential response of the liver to bile acid treatment in a mouse model of Niemann-Pick disease type C” **reports the effect of bile acid treatment on the liver pathology in a mouse model of NPC disease. Although, NPC is primarily considered a neurodegenerative disease, defects in systemic organs including liver have been seen in many patients. The authors previously reported suppression of cytochrome P450 detoxification system in the liver of NPC mice and limited set of human samples. Cytochrome P450 enzymes are also involved in the synthesis of bile acids from cholesterol. The authors hypothesize that exogenously supplied bile acid could potentially alleviate liver disease in NPC. In the study, they used a mouse model to test the hypothesis. The study comes from a lab that holds expertise in NPC and actively advancing the NPC research since last many years. The study was elegantly designed, results are clearly presented and the manuscript is well written and discussed. The conclusions drawn are based on the results reported. The manuscript is suitable to be published in the current form, however if the authors addressed the following comments, it would further enhance the quality of this publication.

  

1. In NPC, many systemic organs including liver show elevated inflammation. One would expect decreased cholesterol burden after UDCA treatment would reduce infiltration of inflammatory cells such as macrophages and neutrophils. It would be nice to have results of a couple of inflammatory marker analysis included in the liver of UDCA treated mice. In addition, it would also be interesting to see how liver enzymes (such as ALT & AST) in mice respond to UDCA treatment.
2. In a separate manuscript (submitted for peer review, ref #10), the authors report the clinical data of four NPC patients treated with bile acid.  The results presented in ref#10 show significant improvement in the liver function in UDCA-treated patients. The submission is a useful extension of this study and shows how findings in an animal model translate to clinics. I would have preferred if both manuscripts were combined. This would allow readers to have a better understanding of the effect of bile acid treatment in NPC in the mouse model as well as in clinics without going through two separate publications.  Nevertheless, this manuscript on its own reports interesting findings.

I have read this submission. I believe that I have an appropriate level of expertise to confirm that it is of an acceptable scientific standard.
